# Replication DNA polymerases, genome instability and cancer therapies

**DOI:** 10.1093/narcan/zcad033

**Published:** 2023-06-28

**Authors:** Juliet D Strauss, Zachary F Pursell

**Affiliations:** Department of Biochemistry and Molecular Biology, Tulane University School of Medicine, New Orleans, 70118 LA, USA; Department of Biochemistry and Molecular Biology, Tulane University School of Medicine, New Orleans, 70118 LA, USA; Tulane Cancer Center, Tulane University School of Medicine, New Orleans, 70118 LA, USA

## Abstract

It has been over a decade since the initial identification of exonuclease domain mutations in the genes encoding the catalytic subunits of replication DNA polymerases ϵ and δ (*POLE* and *POLD1*) in tumors from highly mutated endometrial and colorectal cancers. Interest in studying *POLE* and *POLD1* has increased significantly since then. Prior to those landmark cancer genome sequencing studies, it was well documented that mutations in replication DNA polymerases that reduced their DNA synthesis accuracy, their exonuclease activity or their interactions with other factors could lead to increased mutagenesis, DNA damage and even tumorigenesis in mice. There are several recent, well-written reviews of replication DNA polymerases. The aim of this review is to gather and review in some detail recent studies of DNA polymerases ϵ and δ as they pertain to genome instability, cancer and potential therapeutic treatments. The focus here is primarily on recent informative studies on the significance of mutations in genes encoding their catalytic subunits (*POLE* and *POLD1*), mutational signatures, mutations in associated genes, model organisms, and the utility of chemotherapy and immune checkpoint inhibition in polymerase mutant tumors.

## Graphical Abstract

**Graphical Abstract:**
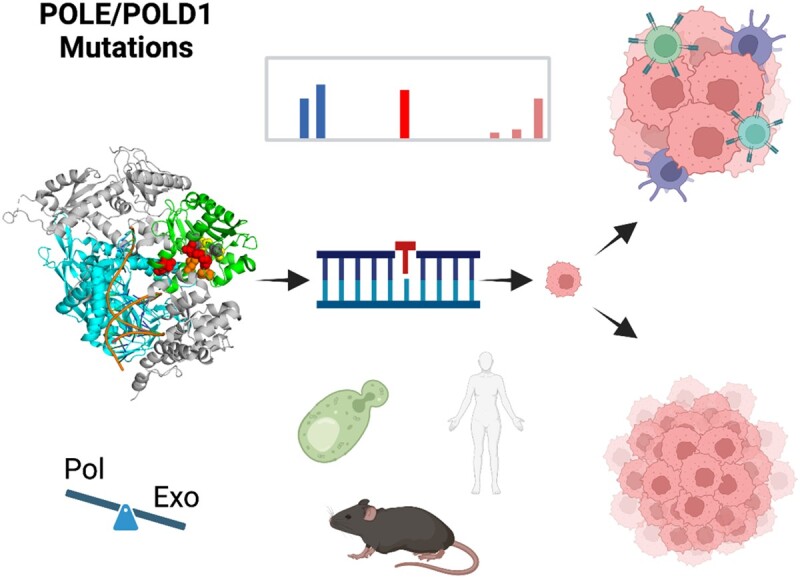

## INTRODUCTION: OVERVIEW OF POLE EXONUCLEASE DOMAIN MUTATIONS IN HUMAN CANCERS

**Figure 1.**
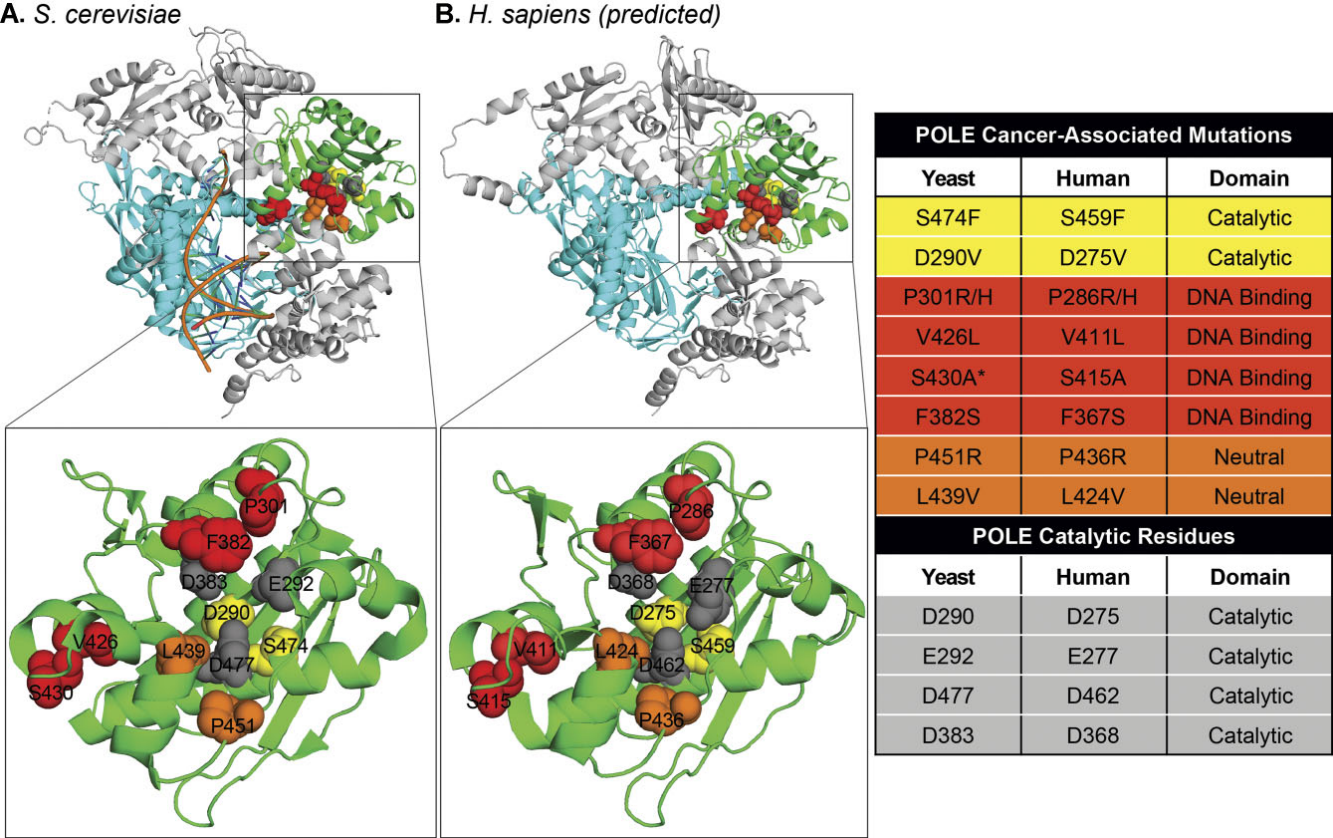
Mapping cancer-associated amino acid residues to POLE structures. The yeast catalytic subunit structure (**A**) is from (38) and the predicted human catalytic subunit structure (**B**) was generated using AlphaFold (39,40). For each enzyme, the exonuclease domain [residues 283–488 in yeast and residues 268–473 in human (41)] is shown in green and the polymerase domain (residues 554–994 in yeast and residues 539–979 in human) is shown in cyan. Magnified images show only the exonuclease domain with cancer-associated POLE mutations (shown as red, yellow, or orange) and catalytic residues (gray) depicted as spheres in the indicated colors. D275 in humans (D290 in yeast) is both catalytic and cancer-associated in humans (*). While not a cancer-associated mutation, S415 in humans (S430 in yeast) is a critical determinant of exonuclease/polymerase activity ratio and is discussed in the main text (*). The yeast structure was solved using D290A/E292A. Figures were generated using PyMOL.

Mutation in the *POLE* gene is now recognized as a driver mutation in human cancers (1–14). Cancer genome sequencing studies uncovered recurrent somatic mutations in *POLE* from somatic human tumors (15–18). Other studies have shown that germline alleles of *POLE* mutations lead to a cancer-prone phenotype (19–26). There are several common threads from these tumor studies that make *POLE* mutant tumors unique. While they can occur in a broad range of cancers, spontaneous *POLE* mutations are particularly enriched in endometrial (9–10%) and colorectal (2–3%) cancers and their tumor mutation burdens (TMBs) are often in excess of 100 mutations per megabase, some of the largest found in all human cancers (15,27). These mutations are found to occur in a unique pattern that has subsequently been found experimentally and computationally to be a composite of three distinct mutation signatures (28–31) (see the ‘Mutation Signatures’ section). The amino acid residues found mutated in *POLE* occur in the exonuclease domain and initial characterization of the mutant enzymes showed that, like catalytic active site mutations, the cancer mutations impaired exonuclease proofreading activity, though to varying degrees (32,33). More precise pre-steady-state kinetic measurements showed that the effects on exonuclease activity ranged over an astounding four orders of magnitude, from ∼2-fold to >44 000-fold (15,34). A major outstanding question is how these mutants with dramatically different effects on catalytic function can all lead to hypermutant tumors with similar mutation signatures. Two residues, P286 and V411, are recurrently mutated, accounting for 50–75% of all *POLE* mutations (35). Structural studies with yeast P301R mutant enzyme, which is orthologous to human P286R, have shown that the arginine creates a positively charged bulge in the exonuclease primer binding groove that prevents the correct orientation for exonucleolytic catalysis (36,37) (Figure 1). The recurrent S459F mutation also drives the unique mutation signature and strongly reduces exonuclease activity (31,34); however, it is not located in a part of the exonuclease domain that would have an obvious effect on activity. The V411L mutation is also well away from the active site and has an unknown effect on structure. The ribbon diagrams (Figure 1) were made using the PDB file from (38) for the yeast enzyme and using AlphaFold (39,40) for the human enzyme. Domains are labeled based on (41).

Increased mutagenesis due to exonuclease domain mutations is not the only way that dysregulated *POLE* can contribute to increased genome instability. Decreasing the protein levels of replication DNA polymerases has previously been shown to increase mutagenesis in yeast primarily in the form of increased DNA polymerase ξ (Pol ξ)-dependent SNVs, increased loss of heterozygosity events and increased single-stranded DNA (ssDNA) (42–46). The increased ssDNA likely results from failure to repair DNA double-strand breaks, since APOBEC3-dependent mutagenesis also increases with increasing ssDNA levels. In cells with mutant Pol δ, this APOBEC3-dependent mutagenesis is elevated on the lagging strand, while it is increased on both leading and lagging strands in Pol ϵ mutants (46). Another interesting difference between Pols δ and ϵ is that low Pol δ levels lead to increased breakpoints at GC-rich sequences, while reduced Pol ϵ does not (45). The authors provide an interesting possible explanation that Pol δ can potentially compensate by replicating these sequences on the leading strand under reduced Pol ϵ levels, but not vice versa. Human cells have also shed light on the effects of reduced Pol ϵ. The Moiseeva laboratory used an auxin-inducible degron mutant allele of *POLE* to show that the CDC45–MCM2–7–GINS complex (CMG) can load in the absence of the Pol ϵ catalytic subunit, but replication forks then quickly collapse (47). CMGs are the functional helicases used to separate double-stranded DNA during replication. The Moiseeva laboratory also found that expression of the C-terminal half of Pol ϵ *in trans* is sufficient to stabilize replication fork firing and suppress ATR activation, though fork progression remains very slow. Decreasing normal Pol ϵ protein levels can also drive replisome dysfunction and human pathologies, including immune deficiency and possibly tumorigenesis. Depletion of *POLE* in humans via homozygous or compound heterozygous mutations that cause splicing defects leads to FILS syndrome, which is characterized by facial dysmorphia, immune deficiency, livedo and dwarfism (48,49). A separate set of patients with IMAGe syndrome also have mutations affecting *POLE* splicing and subsequent protein levels (50). This syndrome is characterized by intrauterine growth restriction, metaphyseal dysplasia, adrenal hypoplasia congenita and genitourinary anomalies in males. These patients have variable immune deficiencies and several even developed lymphomas, including a pediatric T-cell lymphoma. Coupled with observations that *Pole4^−/−^* mice, which lack a small subunit of Pol ϵ (*Pole4*) that helps facilitate holoenzyme processivity, develop increased lymphomas (51), these observations suggest a possible link between *POLE* depletion and tumor development.

## POLD1 MUTATIONS IN CANCER

Altering amino acids in the exonuclease active site of Pol δ has long been known to induce a mutator phenotype in yeast (7,52–55) and to increase cancer mortality in mice (4,56). Mutations in the polymerase domain that decrease replication fidelity and ribonucleotide discrimination (8,57,58) have also been shown to increase cancer mortality in mice (5). In humans, there are far fewer cancer driver mutations in *POLD1* than in *POLE*. The reasons for this relative lack remain unclear, but some clues have emerged. Unlike in *POLE*, where the overwhelming majority of cancer mutations are located in the exonuclease domain, *POLD1* mutations occur in the exonuclease and polymerase domains (15,16), which is the location of the most recurrent *POLD1* mutation, R689W (15,59,60). R689W also occurs in the DLD-1 cell line, which is a widely used epithelial-like colorectal cancer (CRC) cell line. This mutation confers a strong mutator phenotype when engineered into a yeast cell line while retaining full exonuclease activity (60). It was subsequently shown to trigger an expansion of dNTP pools that exacerbates the mutator effect (61). Mutations in the *POLD1* exonuclease domain do exist in human tumors and also have a high TMB (15,16,20). The first reported *POLD1* exonuclease domain mutation, S478N, was seen inherited in a family with predisposition to CRC (20). The same study identified the P327L mutation, which is functionally analogous to *POLE* P286L, in a different patient with CRC tumors, highlighting the conserved nature of this critical proline residue.

One possible partial explanation for the relative lack of *POLD1* mutations in cancer may be the role for Pol δ in what has been called ‘proofreading *in trans*’ (62) or ‘extrinsic proofreading’ (63). Pol δ can proofread DNA synthesis errors made by any of the three replication DNA Pols (α, δ or ϵ), while Pol ϵ can only proofread its own replication errors. In addition to this ability to proofread replication errors, Pol δ exonuclease activity also plays critical roles in mismatch repair (MMR) and Okazaki fragment maturation (64). Mutations that compromise Pol δ exonuclease activity might thus prove catastrophic during tumor development by allowing the accumulation of mutations from DNA synthesis errors made by all three replication DNA polymerases instead of only failing to correct its own errors. It may then be that *POLE* and *POLD1* mutations arise stochastically at similar rates, but the subsequent reduced fitness and decreased viability of *POLD1* mutants give the later appearance of a smaller number of *POLD1* tumors. Of course, this has not been formally tested and other explanations are possible.

## MUTATION SIGNATURES

**Figure 2.**
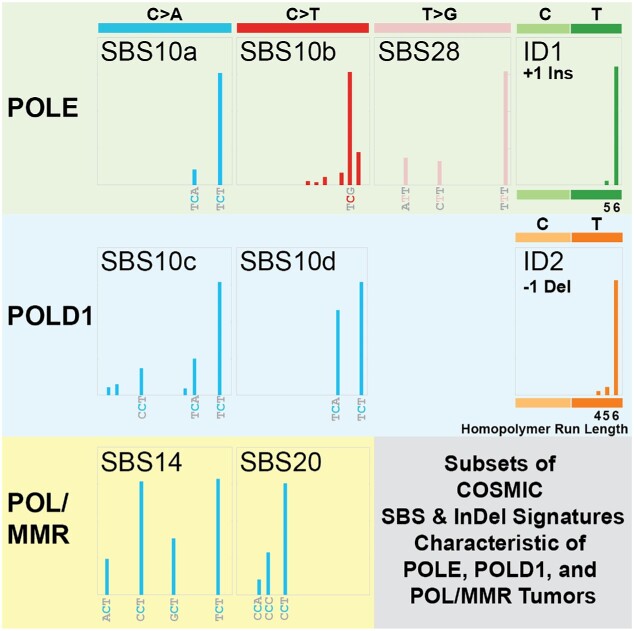
Subsets of COSMIC SBS and indel mutation signatures characteristic of POLE, POLD1 and POL/MMR tumors. For each indicated COSMIC mutation signature (e.g. SBS10a, SBS10c, ID1, etc.) (28), a schematic of its defining trinucleotide mutations is shown alongside the type of DNA polymerase mutation most commonly associated with it (POLE, POLD1 or POL/MMR). The specific trinucleotide or homopolymeric run length is indicated.

*POLE* mutant tumors with functional MMR have a unique mutation signature that was initially categorized as a single mutation signature defined by three distinct trinucleotide context mutations: TCT>TAT transversions, TCG>TTG transitions and TTT>TGT transversions (65). These three mutations have since been computationally resolved into three distinct COSMIC mutation single base signatures (SBSs): 10a, 10b and 28, respectively (28) (Figure 2). *POLE* mutant tumors lacking MMR give rise to a separate mutation signature, SBS14, characterized by multiple C>A transversions, primarily in NCT contexts (28,30). A unique insertion/deletion (indel) signature was later discovered in *POLE* mutant tumors, consisting of an insertion of a single +A in a run of 5–10 consecutive As (66). Around this same time, COSMIC defined two indel signatures, ID1 and ID2 (28). ID1 is defined by insertion of a single T in homonucleotide runs of T ≥ 6 and ID2 and is essentially the same as what was observed in POLE mutant tumors (66). ID2 is defined by loss of a single T in homonucleotide runs of T ≥ 6. Samples that contained large numbers of ID1 or ID2 indels also had SBSs associated with loss of MMR alone (e.g. SBS6, SBS15) or in addition to POLE or POLD1 mutations (e.g. SBS14, SBS20) (28). Critically, the mechanisms that drive these different mutation spectra are poorly understood. The relative abundance of SBS10b TCG>TTG transitions has been shown to be highly variable in cells and tumors (31) and some have speculated that they are in fact due to replication past deaminated 5-methylcytosines in CpG sequences (29,67).

*POLD1* mutation signatures have been more difficult to identify, in large part due to the much smaller number of *POLD1* tumors and, therefore, fewer Pol δ-dependent mutations. Sequencing normal cells from *POLE* and *POLD1* carriers showed that there is increased Pol-dependent mutation burden in intestinal crypt stem cells, sperm and embryonic tissue. While these carriers do have increased risk of developing cancer, they do not demonstrate early aging phenotypes (68). This same study identified two *POLD1*-associated mutation signatures, SBS10c and SBS10d. SBS10c is characterized by enriched C>A transversions at TCT and more moderate peaks for CCT/TCA, while SBS10d is dominated by C>A at TCA/TCT. The similarities of the TCT>TAT transversions between *POLD1* SBS10c/d to those in *POLE* SBS10a may hint at a common mechanism shared by *POLE* and POLD1 tumors, but the differences in the moderate peaks in POLD1 SBS10c/d and the T>G transversions in SBS28 point to likely separate and distinct mechanisms. What underlies both the similarities and differences remains to be identified.

## ASSOCIATED MUTATIONS

Mutations in *PTEN* and *TP53* have been observed to be enriched in *POLE* mutated tumors. Since nearly every open reading frame in these tumors has at least one mutation, a challenge has been to determine the functional significance, if any, of these associated mutations. *PTEN* has a well-defined function as a negative regulator of the PI3K-AKT pathway via its lipid phosphatase catalytic activity (69–71). Loss of *PTEN* function is canonically thought to drive tumor progression by generating sustained pro-growth signals through downstream targets. However, *PTEN* also has noncanonical tumor suppression roles that operate through a variety of pathways, including regulating and maintaining genome stability, heterochromatin states, replisome firing and coordination, and checkpoint control (72,73). *PTEN*’s role in genome stability is mediated through nuclear translocation, direct interaction with replication and repair factors, and protein phosphatase activities (74,75). *PTEN* directly colocalizes with replication foci even when cells are challenged with hydroxyurea (HU) (76). Disrupting *PTEN* interactions with replisome and fork rescue components (e.g. PCNA, MCM2, RPA1 and RAD51) triggers stalled forks and increases replication stress, suggesting that *PTEN* plays a critical role in fork stabilization and restart after replication stress (76,77). Directly related to *POLE*, *PTEN*-deficient cells show increased spontaneous fork stalling and decreased fork restart when challenged with HU or aphidicolin (78). Critically, *PTEN*-deficient cells show reduced fork-associated RAD51, PCNA and CHEK1 after HU treatment. Addressing how *POLE* mutant cells can deal with loss of these genome stabilizing roles and what other mutations are associated with *POLE* tumors will help better understand the functions of the cancer mutations.

## MODEL SYSTEM DATA

Since Pols ϵ and δ are conserved in all eukaryotes, the single-cell yeasts *Saccharomyces cerevisiae* and *Schizosaccharomyces pombe* have long been excellent model systems for studying their functions (6–9,11,53,60,64,79–88). More recently cancer mutations have been modeled in yeast to study their effects on mutagenesis (24–26,36,60,61,67,89). One study that performed a comprehensive comparison of multiple mutant alleles showed over two orders of magnitude difference in mutation rate depending on the particular mutation examined (25). The P286R and S459F alleles (residues are noted by their orthologous human amino acid except where specifically noted) caused 150- and 30-fold increases in mutagenesis, respectively, while the V411L allele was no different than wild type in haploid yeast. This large difference is intriguing because P286R and V411L are the two most frequent *POLE* mutations in cancer and tumors from both have high TMB. The same study also showed that very modest mutator alleles can cause synergistic increases when combined with defects in MMR.

Soriano *et al.* recently reported using *S. pombe* to model *POLE* mutations (67). As in *S. cerevisiae*, the P286R mutation was a strong mutator. The SBS10a hallmark TCT>TAT transversions predominated, with a minor fraction of SBS28-like NTT>NGT transversions. An interesting, though subtle difference was the lack of a strong TTT>TGT seen in human and mouse tumors. The largest difference, however, was the complete absence of SBS10b TCG>TTG transitions, which the authors ascribe to the lack of cytosine methylation in *S. pombe*. They also found that *S. pombe* cells with mutated P286R are sensitive to a variety of DNA damaging agents, including ultraviolet (UV) light, methyl methanesulfonate and bleomycin, suggesting a possible defect in replication fork progression rather than the sensitivity being due to a particular DNA lesion or adduct. The P286R cells are also modestly resistant to HU, which decreases dNTP concentrations, but synthetic lethal with mutations that increase overall dNTP levels. The presence of the P286R mutation did not raise dNTP levels by itself, both in *S. pombe* (67) and in *S. cerevisiae* (37). They also found that knocking out Pol κ, a specialized translesion (TLS) DNA polymerase, reduced P286R-dependent mutagenesis by 6–7-fold, implicating roles for TLS-dependent synthesis and polymerase switching in *POLE* mutagenesis. Loss of Pol ξ, however, had no effect on mutagenesis in either yeast (37,67).

Recent studies using yeast have also provided insight into the possible functional consequences of V411L (24,90), the second most common *POLE* mutation in cancer. This residue is in a hairpin that is structurally at a distance from the exonuclease and polymerase active sites. Previous observations from different organisms have shown this mutation to have unique properties when compared to mutations residing within the defined exonuclease domain, like P286R (36). The V411L mutant allele has previously been shown to be a very weak to nonmutator in haploid yeast (25). Human tumors with V411L have slightly lower, yet still high overall, TMB than do tumors with P286R, and have weaker presence of *POLE* mutation signatures (31). The human enzyme with V411L was previously shown to have only modest effects on exonuclease activity (33). Pellicanò *et al.* showed that phosphorylation of the DNA Pol ϵ catalytic subunit in yeast is critical for maintaining the balance between DNA synthesis and strand resection induced by replication fork stalling (90). The phosphorylated residue, S430 in *S. cerevisiae*, is adjacent to the β-hairpin loop that contains V411 (V426 in *S. cerevisiae*) that mediates polymerase/exonuclease switching in B-family DNA polymerases (91,92). Upon fork stalling, the nascent strand partitions to the exonuclease active site, driving resection. If left unchecked, this resection could hasten eventual fork collapse. They showed that when the S430 residue is phosphorylated, partitioning to the exo site is blocked, thus reducing strand resection and helping maintain fork stability. The S430A mutant, which cannot be phosphorylated, also cannot block this partitioning. As a result, Pol2-S430A cells accumulate large amounts of stress-induced ssDNA and are highly sensitive to HU, which can be rescued by inactivating exonuclease activity.

Barbari *et al.* showed that the V411L mutant is a strong mutator when MMR is inactivated (24). They also showed that the Pol ϵ enzyme with V411L mutation is an even more hyperactive polymerase on a hairpin-containing template *in vitro* than the P286R mutant (24). This activity is further increased when the hairpin template has a primer terminal mispair. Different exonuclease domain mutations that disrupt partitioning from the polymerase to the exonuclease site also increase mispair extension *in vitro* and are strong mutators *in vivo* (93). A new 68 amino acid motif adjacent to the catalytic core was found in yeast with homology to the same region in human *POLE* (94). When mutations present in this motif in human cancers were engineered into yeast Pol ϵ, gross chromosomal rearrangements increased while having no effect on single nucleotide variant mutagenesis. These mutants also have reduced replication fork progression that was later found to be suppressed by ablating exonuclease activity (95). Taken together, recent work has pointed to both intrinsic and extrinsic factors that contribute to drive the unique mutagenesis found in *POLE* cancer mutants. One recurring theme is the importance of the balance between DNA synthesis and exonuclease activity. Tipping this balance in either direction not only has consequences for immediate replication fidelity, but also appears to drive dysregulation at the replication fork. To what extent this underlies the ability of *POLE* or *POLD1* mutants to drive tumor development remains to be explored.

## IMMUNE THERAPY APPROACHES

Immune checkpoint inhibitor (ICI) therapies have proven successful in treating solid tumors with high TMBs, especially when the high TMB is a result of MMR deficiencies or UV-induced DNA damage (96,97). Polymerase proofreading-deficient tumors have been a potential ICI target based largely on their high TMB and high degree of immune infiltrates, particularly CD8^+^ cytotoxic T-cell lymphocytes, as compared to microsatellite stable (MSS) tumors with wild-type *POLE* (98–100). One early report of ICI therapy efficacy in polymerase-mutated cancer was from two siblings with multifocal glioblastoma multiforme (101). The siblings both had biallelic MMR disorder and so completely lacked MMR, with whole exome sequencing showing that the glioblastoma tumors had acquired spontaneous pathogenic mutations in *POLE*. Both siblings had durable and prolonged responses to single-treatment nivolumab (α-PD-1). In another early case report, a 57-year-old woman with recurrent, multi-therapy-resistant, stage III endometrial cancer with a *POLE* P286R mutation showed sustained response to α-PD-1, including regression of abdominal, retroperitoneal and pelvic deposits 7 months after follow-up (102). Prostate tumors with *POLE* mutations and MSS have also been reported as having a durable response to α-PD-1 treatment after failure of chemotherapy and surgery (103).

A more recent study used a prospective cohort of *POLE* mutated solid tumors to test efficacy of single-treatment α-PD-1 (104). They restricted enrollment to specific criteria that have been used to distinguish driver from passenger *POLE* mutations (nonsynonymous *POLE* exonuclease mutation, high TMB, evidence of *POLE* mutation signature) (31), with the addition of including high immune infiltrate. They identified 12 tumors (5 CRC, 6 endometrial and 1 glioblastoma) with bona fide functional *POLE* mutations. Durable and significant responses were seen in seven patients, and the best responding patients were those with high TMB, the highest fraction of POLE signature mutations and higher immune infiltrates as measured by immune deconvolution of bulk RNA sequencing data. Responses were also observed exclusively in MMR-proficient/MSS tumors. A separate study of 500 colorectal tumors included seven *POLE* tumors that had generally higher levels of tumor infiltrating lymphocytes (TILs) (105), which is consistent with the high TMB in these tumors. However, the presence of a *POLE* mutation with high TMB is not sufficient to attract TILs as *POLE* tumors had both TIL-high and TIL-low subsets. As this study did not provide the *POLE* variants, it would be interesting to determine whether there are any particular variants, possibly V411L, that might be overrepresented in the TIL-low set.

The same study went further, using structural modeling to ask whether responders could be divided based on the spatial position of mutated amino acid residues. They describe three distinct spatial locations, exo catalytic site (e.g. P286), DNA binding site (e.g. S459) and neutral site (e.g. R446) (Figure 1), and show that mutations mapping to the catalytic and DNA binding sites generally respond to α-PD-1 better than neutral site mutations. They also show that higher allelic frequencies of *POLE* mutations generally respond better, arguing that subclonal, and likely later occurring, mutations may have attenuated responses. One interesting observation is from the V411L tumors. While only three were in this cohort, they defy easy categorization based on the authors’ criteria. While two V411L patients showed ongoing partial responses, the patient with the largest increase from baseline in tumor size and with progressive disease (RECIST v1.1) while on treatment had a V411L mutation. Since the TMBs in all tumors were sufficiently high to generate the significant number of neo-antigens predicted to drive response, simple abundance of neo-antigens is unlikely to be the cause of the variability in clinical response. The proportion of *POLE*-related SBS does differ, however, between patient groups. The *POLE*-related fraction of total SBS is lower for nonresponders (1–60%) and higher for responders (>60%). Given the recent insights into how V411L mutants may have somewhat different mechanisms than bona fide exonuclease domain mutations, it is worth considering whether this may be at least partly responsible for the varying response in these tumors.

A retrospective analysis of over 14 000 patients at MD Anderson Cancer Center found a significant benefit for the 68 patients with pathogenic *POLE* variants when treated with ICI (106). Median overall survival was 29.5 months for *POLE* patients with pathogenic variants (defined in the study as being annotated by InterVar or ClinVar and having peer-reviewed citations), versus 11.6 months for nonpathogenic or uncertain variants. Agreeing with an earlier, smaller cohort of endometrial cancer patients (1), they show that tumors with pathogenic *POLE* variants confer a survival benefit regardless of treatment. Median survival was not reached for these patients, versus 6.4 years for those with nonpathogenic variants. There is interesting future work to be done on *POLE* variants of unknown significance, as 38% had a positive response to ICI therapy. Since the number of *POLD1* mutant tumors in humans remains small, there has yet to be a larger scale measurement of ICI response in these tumors. However, a study that used CRISPR to separately engineer different *POLE* and *POLD1* mutations into two different mouse tumor cell lines found that ICI sensitivity was significantly improved in those syngeneic *POLE* and *POLD1* tumors (107). This raises the possibility that the benefits of ICI to *POLD1* tumors may be similar to what is seen for *POLE* tumors.

In examining how *POLE* tumors respond to chemotherapy treatments, there are possible clues as to how *POLE* mutations may affect cellular responses to DNA damage. There are now multiple case reports of *POLE* patients displaying resistance to different chemotherapeutic treatments, including platinum drugs, topoisomerase-1 inhibitors and 5-fluorouracil (5-FU) (102,103,108). In one such case of a chemotherapy-treated patient with progressive disease, the *POLE* F367S exonuclease domain mutation was identified, and the patient was then switched to pembrolizumab, another α-PD-1 ICI. The patient subsequently had a complete response that persisted past 2 years (108). Several groups have shown resistance to carboplatin, cisplatin, 5-FU, doxorubicin and etoposide for *POLE* mutant cells in culture (109,110). Future work will likely uncover what may be driving this increased resistance to DNA damaging agents: something intrinsic to *POLE* mutants, increased incidence of inactivating mutations in repair and/or signaling pathways in TMB-high *POLE* tumors or some other feature.

## SUMMARY

The identification and characterization of cancer mutations in replication DNA polymerases have not only helped our understanding of mutagenesis and tumor development but also helped increase our understanding of basic replisome functions. There is much still to be learned, however. After a decade of study using multiple systems, we still do not fully understand the basic mechanism behind the distinct mutation signatures in *POLE* and *POLD1* tumors. And now the observations that many, but not all, of the highly mutated *POLE* and *POLD1* tumors respond to immune checkpoint therapies are poised to help us predict when certain therapies may be beneficial and when they may not. The next decade is likely to bring fresh insights into how the replisome is intimately linked to genome instability, cancer development and predicting therapeutic outcomes in patients.

## SOME KEY OUTSTANDING QUESTIONS

- How do different POL mutations that show dramatically different effects on exonuclease activity result in such similar mutation signatures in tumors and cells?
- What causes the relative lack of POLD1 mutations in tumors overall, but particularly in the exonuclease domain?
- What underlies the similarities and differences in the various polymerase- and MMR-associated mutation signatures?
- How does the balance between polymerase and exonuclease activities contribute to mutagenesis and cancer?
- What factors contribute to driving POL tumor development and mutation signatures?
- What are the key determinants driving response (or lack thereof) to immune checkpoint therapies in POL tumors?

## DATA AVAILABILITY

No new data were generated or analyzed in support of this research.
